# Deficient and Null Variants of SERPINA1 Are Proteotoxic in a *Caenorhabditis elegans* Model of α1-Antitrypsin Deficiency

**DOI:** 10.1371/journal.pone.0141542

**Published:** 2015-10-29

**Authors:** Erin E. Cummings, Linda P. O’Reilly, Dale E. King, Richard M. Silverman, Mark T. Miedel, Cliff J. Luke, David H. Perlmutter, Gary A. Silverman, Stephen C. Pak

**Affiliations:** 1 Department of Pediatrics, University of Pittsburgh School of Medicine, Children's Hospital of Pittsburgh of UPMC, Pittsburgh, Pennsylvania, United States of America; 2 Department of Cell Biology and Molecular Physiology, University of Pittsburgh School of Medicine, Pittsburgh, Pennsylvania, United States of America; Centro de Investigación en Medicina Aplicada (CIMA), SPAIN

## Abstract

α1-antitrypsin deficiency (ATD) predisposes patients to both loss-of-function (emphysema) and gain-of-function (liver cirrhosis) phenotypes depending on the type of mutation. Although the Z mutation (ATZ) is the most prevalent cause of ATD, >120 mutant alleles have been identified. In general, these mutations are classified as deficient (<20% normal plasma levels) or null (<1% normal levels) alleles. The deficient alleles, like ATZ, misfold in the ER where they accumulate as toxic monomers, oligomers and aggregates. Thus, deficient alleles may predispose to both gain- and loss-of-function phenotypes. Null variants, if translated, typically yield truncated proteins that are efficiently degraded after being transiently retained in the ER. Clinically, null alleles are only associated with the loss-of-function phenotype. We recently developed a *C*. *elegans* model of ATD in order to further elucidate the mechanisms of proteotoxicity (gain-of-function phenotype) induced by the aggregation-prone deficient allele, ATZ. The goal of this study was to use this *C*. *elegans* model to determine whether different types of deficient and null alleles, which differentially affect polymerization and secretion rates, correlated to any extent with proteotoxicity. Animals expressing the deficient alleles, Mmalton, Siiyama and S (ATS), showed overall toxicity comparable to that observed in patients. Interestingly, Siiyama expressing animals had smaller intracellular inclusions than ATZ yet appeared to have a greater negative effect on animal fitness. Surprisingly, the null mutants, although efficiently degraded, showed a relatively mild gain-of-function proteotoxic phenotype. However, since null variant proteins are degraded differently and do not appear to accumulate, their mechanism of proteotoxicity is likely to be different to that of polymerizing, deficient mutants. Taken together, these studies showed that *C*. *elegans* is an inexpensive tool to assess the proteotoxicity of different AT variants using a transgenic approach.

## Introduction

The most abundant proteinase inhibitor in the circulation and extracellular fluids is α1-antitrypsin (AT)/SERPINA1. AT is an irreversible inhibitor of neutrophil elastase and other serine granule peptidases, such as proteinase 3 and cathepsin G [1–4]. Based on these biochemical features, congenital deficiencies of AT (ATD), in part, lead to disorders associated with excessive proteolysis [5]. The classical form of ATD, an autosomal recessive disorder due to the Z (E342K) mutation, leads to excessive proteolytic digestion of the pulmonary extracellular matrix and emphysema. This lung injury is a direct consequence of markedly decreased circulating levels of the inhibitor and is a classical loss-of-function disease phenotype. However, the Z mutation also leads to a gain-of-function disease phenotype initiated by impaired protein folding and domain swapping between mutant serpin molecules (ATZ). This process leads to the toxic accumulation of monomers, oligomers, polymers and higher-order insoluble aggregates in the ER of hepatocytes [6]. These aggregates appear as diastase-resistant and periodic acid Schiff (PAS)^+^ inclusions in histopathological specimens [7–9]. The proteotoxicity of ER-retained ATZ ultimately leads to cirrhosis, fibrosis and hepatocellular carcinoma in some patients [7, 9–14].

ATD associated with the homozygous Z mutations (Pi*ZZ genotype) occurs in ~ 1/2,000 live births in Northern European and North American populations, and is the most common genetic cause of liver disease leading to pediatric liver transplantation [9, 10]. Approximately 10–15% of Pi*ZZ patients develop severe liver disease over the first 2 decades of life [15, 16], and some studies suggest ~30–40% develop clinical evidence of injury later in adulthood [14, 17–19]. Several studies also have suggested that heterozygotes (PiZM) exposed to other types of liver injury, such as hepatitis B and C, may be more susceptible to developing end-stage liver disease [20–24].

ATD was first described in 1963, as the absence of the α_1_ band on serum protein electrophoresis [25]. Many AT variants were named according to their migration pattern observed after isoelectric focusing, with variants A-L running faster, and N-Z running slower than the wild-type “M” allele (ATM) [26]. Although the majority of ATD patients (~95%) are of the Pi*ZZ genotype, [27, 28], >120 different AT mutations have been reported [29–32]. In general, AT coding variants fall into 3 major categories [33]. First are the normal alleles, such as M1, M2, M3 and M4, which yield normal circulating levels of fully active inhibitor. Second are the deficient alleles, such as Z and Mmalton, which are caused mostly by missense mutations or small deletions. Protein synthesis yields nearly full-length misfolded proteins that are retained in the ER. Plasma levels are <20% of normal and serpin inhibitory activities are variably diminished [29, 34, 35]. Third are the null alleles, such as Hong Kong (NHK), which result from nonsense mutations or frameshifts leading to premature stop codons. Depending on exon position, the stop codon triggers nonsense-mediated decay or the formation of truncated proteins that are efficiently degraded intracellularly. Plasma levels are undetectable (<1% of normal) [32, 36]. There is a fourth category of extremely rare variants, the dysfunctional alleles [33]. The best-known example in this group is Pi*Pittsburgh (M358R), which functions as a constitutively active (heparin independent) form of antithrombin [37, 38]. Since, many deficient and null variants have not been compared side-by-side in a single model system, the ability to predict disease risk due to any form of a toxic gain-of-function phenotype, has been difficult to assess. This difficulty is compounded in population-based studies due to their rare occurrence, variable penetrance and expressivity and the presence of disease modifiers [30, 35, 39, 40].

The pathogenicity of different human disease related alleles could be ascertained more clearly by examination of gene function in isogenic strains. Transgenic mice, such as the PiZ mouse (expressing ATZ) proved to be an invaluable resource in demonstrating conclusively that liver disease is due to a gain-of-function phenotype, and not the loss of ATM expression [41]. However, we are unaware of any other mouse models examining the pathogenicity of other AT variants. Since the generation of multiple transgenic mouse strains is expensive and time consuming, we sought to determine whether a simpler transgenic approach with higher processivity might be available to study the cellular proteotoxicity associated with different AT variants.

We recently developed a transgenic model of ATZ-induced cellular injury in *C*. *elegans*, one of the premier organisms for modeling the molecular, genetic and cellular basis of human diseases [42]. Remarkably, transgenic animals expressing a secreted form of GFP (sGFP) fused to ATZ (sGFP::ATZ), but not the wild-type protein (ATM), recapitulate the ER-retention phenotype and acquire intracellular inclusions (dilated ER cisterna) similar to those present in ATD hepatocytes [43]. Animals also showed proteotoxic injury manifest by slow growth, small brood sizes and decreased longevity; phenotypes observed in transgenic mouse models [41]. As in humans and mice, the conventional (macro)autophagy, and to a lesser extent, ER-associated degradation/ubiquitin proteasomal pathways (ERAD-UPS) eliminate misfolded ATZ [43].

The objective of this study was to use our *C*. *elegans* model system to determine whether different types of AT deficient alleles, which differentially affect polymerization and secretion rates *in vitro* [44, 45] and cell lines [46], respectively, correlated to any extent with proteotoxicity *in vivo*. For comparison to these deficient alleles, we included null variants, which heretofore were not associated with any degree of hepatic proteotoxicity [36, 47, 48]. The deficient alleles were proteotoxic and correlated with their ability to induce disease clinically. Surprisingly, we also found that the null mutations were proteotoxic, but to a much lesser degree than the aggregation-prone deficient mutants. These findings suggest that any AT variant leading to a non-native state has the potential to generate a proteotoxic gain-of-function phenotype.

## Materials and Methods

### *Caenorhabditis elegans* strains and culture conditions

Animals were cultured at 22°C on nematode growth medium (NGM) seeded with *E*. *coli* strain OP50, unless otherwise specified. *Caenorhabditis elegans* strain N2 was obtained from the *Caenorhabditis* Genetics Centre (CGC), http://www.cbs.umn.edu/CGC/. Generation of transgenic P_*nhx-2*_sGFP::ATM and P_*nhx-2*_sGFP::ATZ expressing lines has been previously described [49].

P_*nhx-2*_ *sGFP*::*Mmalton*, P_*nhx-2*_ *sGFP*::*Siiyama*, P_*nhx-2*_ *sGFP*::*ATS*, P_*nhx-2*_ *sGFP*::*NHK*, P_*nhx-2*_ *sGFP*::*Saar* expression constructs were generated by site-directed mutagenesis (QuikChange XL Mutagenesis Kit, Stratagene) using P_*nhx-2*_ *sGFP*::*ATM* as the template. A complete list of mutagenesis primers used in this study is shown in S1 Table. Transgenic animals were generated by microinjecting young adult animals with P_*nhx-2*_ *sGFP*::*AT*(mutant) and P_*myo-2*_ *mCherry* plasmids at a final concentration of 80 ng/μl. Transgenic lines maintaining stable extrachromosomal arrays were integrated by irradiation. Integrants were outcrossed with N2 a minimum of 6 times to remove background mutations. S2 Table contains complete list of strains used in this study.

### RNAi Experiments

All RNAi experiments were performed by using the feeding method as described [43, 50]. Briefly, 50 mL of LB media, containing 100 μL/mL ampicillin, was inoculated with 1 ml of an overnight bacterial culture containing the RNAi plasmid of interest. The culture was incubated in a 37°C shaker, until the optical density at 600 nm was 0.5. After placing on ice briefly to halt growth, the culture was induced for 2 hours by adding IPTG to a final concentration of 1 mM. Following the induction, bacteria were pelleted by centrifugation at 5000 rpm for 10 minutes. The pellet was resuspended in 5 mL LB media containing 100 μg/mL ampicillin. The concentrated RNAi culture was seeded onto 100 mm NGM plates, containing IPTG and ampicillin. The plates were incubated at 37°C overnight prior to use. For a typical RNAi experiment, approximately 100 L4 worms were placed onto the respective plates containing the induced RNAi culture and incubated at 22°C. After 48 hours, animals were assessed for the desired phenotype. In some experiments, F1 progeny were transferred to fresh RNAi plates to assess the desired phenotype in the next generation.

For quantification of sGFP::AT expression, we acquired images of the animals using the ArrayScan V^TI^ HCS Reader (Cellomics, ThermoFisher, Pittsburgh, PA, USA) and performed data analysis using the SpotDetector BioApplication as described [49]. Experiments were repeated 6 times and a representative experiment is shown in the figures. Error bars represent the SD of at least two replicates within the experiment.

### Immunoblotting

Western blotting analyses of whole worm lysates were performed under denaturing and reducing conditions. First, well-fed animal populations were washed from 15 cm NGM plates and pelleted by centrifugation. The worm pellets were resuspended in 2X SDS loading buffer and boiled for 5 minutes. Once cooled, the samples were sonicated briefly to complete the lysis. After centrifugation at 12,000 x g for 3 minutes the supernatant was transferred into a fresh tube. For SDS PAGE analysis, 10 μL of each sample was loaded into each well of a 10% Criterion TGX precast gel (Bio-Rad, Hercules, CA, USA) and run at 250V for approximately 30 minutes. The protein was then transferred to a nitrocellulose membrane at 100 V for 2 hours using the Criterion protein transfer system. The membrane was blocked overnight in blocking buffer (TBST: 50mM Tris HCl, 150mM NaCl, 0.05% Tween 20, 5% milk powder) at 4°C. Blots were incubated in primary and secondary antibodies at room temperature for 1 hour each. GFP::AT fusion bands were detected using a rabbit anti-GFP polyclonal antibody (Sigma-Aldrich) followed by a bovine anti-rabbit-HRP conjugated antibody (Santa Cruz Biotechnology). α-tubulin bands were detected using a mouse anti-α-tubulin monoclonal antibody (Sigma-Aldrich) followed by bovine anti-mouse-HRP conjugated antibody (Santa Cruz Biotechnology). Actin bands were detected using a mouse anti-actin monoclonal antibody (Millipore) followed by bovine anti-mouse-HRP conjugated antibody (Santa Cruz Biotechnology). All bands were visualized using Luminata Forte Western HRP Substrate (Millipore). Densitometric quantification was performed using the open source ImageJ software (http://imagej.nih.gov/ij/) [51]. AT protein band was normalized using the β-actin or α-tubulin controls. Data were collected from 4 independent experiments.

### Quantitative Real time PCR (qRT-PCR)

Approximately 1000 L4 animals were transferred to a fresh 15cm NGM plate and allowed to grow for 2 days. mRNA was extracted using TRIzol (Life Technologies, NY) according to the manufacturer’s protocol. Three biological replicates were collected for each AT variant line. The mRNA was quantified (NanoDrop, Thermo Scientific), and 1 μg was treated with DNAse I (Sigma-Aldrich) to remove genomic DNA and subject to first strand cDNA synthesis (Superscript III, Invitrogen). A no RT control was also prepared for each line. The cDNA was then diluted to 50 ng/well and subject to qRT-PCR (SensiMix SYBR Hi-ROX, Bioline, ABI Prism, Applied Biosystems), using primers designed to detect the GFP (Forward 5’-AGCACTGTAAGAAGCTGTCC-3’, Reverse 5’-TCCAGTAATGGACAGTTTGG-3’). Three experimental replicates were prepared for each sample. The Ct count was then normalized to house-keeping gene, *rpl-32*, and expressed as fold change of ATM, calculated by the 2(-Delta Delta C(T)) method. Error bars represent the SEM from 3 biological replicates.

### Microscopic imaging

For image acquisition, approximately 15 worms were transferred onto a 35 mm MatTek glass bottom culture dish (MatTek, Ashland, MA, USA) containing a 6 μL drop of 50 mM sodium azide (NaN_3_). Confocal images were collected using a Leica TCS SP8 microscope. GFP fluorescence was illuminated using a 488 nm argon laser line and red fluorophors with a 561 nm solid state laser with either a 20x 0.6NA Apochromat air objective, or a 40x 1.3NA oil Apochromat CS2 objective. Images were captured using a spectral NyD detector. DIC images were collected using a transmitted light detector and the 488 nm argon laser line. Confocal images were acquired using LAS AF software (Leica Microsystems) and visualized, rendered, and analyzed using Volocity Software (Perkin Elmer). For quantification of aggregates, images were captured with the 40x objective, from the vulva to tail region of each animal, and analyzed using the Volocity software. Approximately 10 worms were imaged for each line and the data represented as a scatter plot. Experiments were repeated three times and representative data shown in the figures. To quantify eggs in utero, 40x DIC images of the uterus were collected. Eggs number was counted manually. Experiments were conducted at least 3 times and data were collected from at least 10 animals per line.

### Lifespan analysis

For lifespan studies, 25 L4 animals were placed onto a 10 cm NGM plate seeded with OP50 and FUDR. Plates were examined each day and the number of alive and dead animals was recorded. An animal was considered dead if it did not respond to gentle touch with a platinum wire pick. Animals that crawled off the plate or could not be accounted for were recorded and censored from the final analysis. Results were plotted as Kaplan-Meier survival curves and statistical significance determined using the Log-Rank (Mantel-Cox) test.

### Post-embryonic development

Worm populations were synchronized for analysis of post-embryonic development through the egg-lay method. Adult worms (n = 25–30) were permitted to lay eggs for 2 hours onto 10 cm NGM plates seeded with OP50. The adult worms were removed and the eggs placed at 25°C to hatch and develop for 48–52 hours. Under these conditions, >90% N2 animals were in the L4-young adult stage. At the end of the incubation period, each plate was examined and the number of animals in the each of the post-embryonic developmental stages (L1, L2, L3, L4 and adult) was recorded. Animals that were in the L1, L2, and L3 larval stages were considered slow growing and were scored as Gro. Animals in the L4 and adult worms were scored as normal.

### Brood size measurements

For determination of brood size, 10 L4 stage animals were placed onto individual 6 cm NGM plates seeded with OP50 to analyze the total progeny produced by each animal. The plates were stored at 22°C for the duration of the experiment. Every day, the original worm was carefully transferred to a new plate, and the progeny counted and recorded. This process continued throughout the reproductive lifespan of the worms (5–7 days). The brood size was the cumulative total number of eggs laid by a single animal. Multiple (>20) animals of the same genotype were assessed to obtain the mean and standard deviation.

### Statistics

Statistical analysis of the data was carried out using Prism (Graphpad Software). Statistical significance between average size of aggregates, relative protein and mRNA, and relative GFP intensity per animal were determined using a two-tailed student's *t*-test. P-value summary: *P<0.05, **P<0.01 and ***P<0.001. ns, not significant. Statistical significance between survival curves of N2 and AT variants were determined using the Log-Rank (Mantel-Cox) Test. P-value summary: **P<0.005 and ***P<0.0001.

## Results

### Expression of human AT variants in *C*. *elegans*

**Fig 1:**
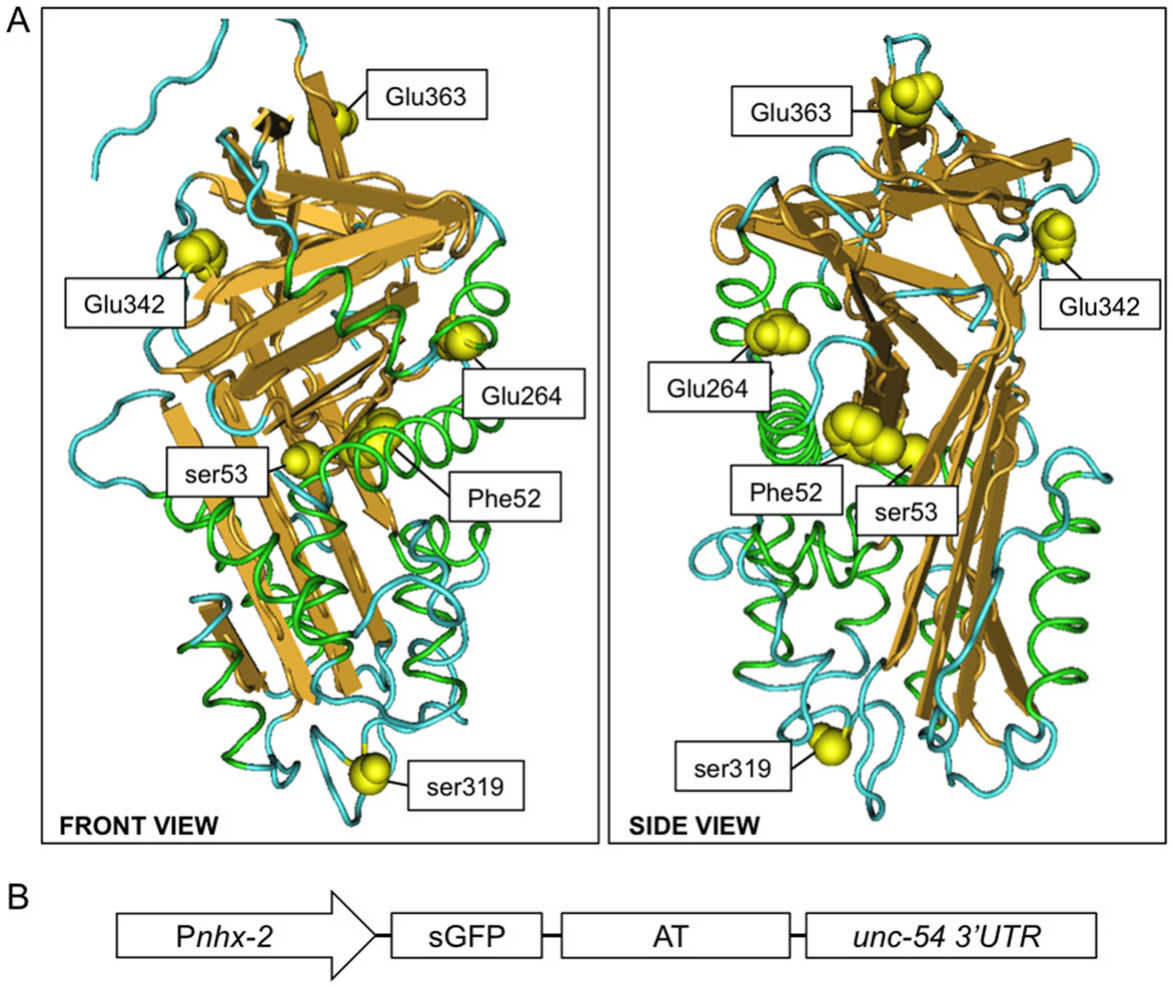
AT variants and transgenic constructs. A ribbon diagram of α1-antitrypsin (PBD, 3NE4) highlighting the positions of the amino acid residues associated with deficient and null alleles (A). A schematic depicting the *C*. *elegans* expression construct (B). P_*nhx-2*_, intestine-specific promoter; sGFP, green fluorescent protein with an N-terminal signal peptide; AT, α1-antitrypsin.

To determine whether different AT variants showed gain-of-function phenotypes comparable to ATZ in *C*. *elegans*, we selected three AT deficient alleles: Mmalton (*F52del*), Siiyama (*S53F*) and S (*E264V*, ATS). The rationale for selecting these AT variants from the >120 that have been reported is based on the nature of the mutation, their physiochemical properties and their clinical presentation. Two of the variants, Mmalton and Siiyama, harbor mutations located on helix B, one of the main structural elements that facilitate the mobility of strands 3A and 4A (i.e., the shutter region) [52, 53] (Fig 1A). Like ATZ, both Mmalton and Siiyama are associated with subtherapeutic plasma levels and hepatic inclusions [52–56]. Both mutant proteins also form polymers [57, 58]. However, there are differences between these aggregation-prone mutants. Mmalton is secreted at a rate of ~twice that of ATZ in *Xenopus* oocytes [57], and has normal inhibitory kinetics with neutrophil elastase [52]. In contrast, ATZ inhibitory activity is ~45% of normal [59]. However, Siiyama, which is secreted more efficiently than ATZ in COS-1 cells, appears to lack inhibitory activity [46, 58]. The mutation in the third deficient variant, ATS, localizes to helix G (Fig 1A). In contrast to Pi*ZZ patients, Pi*SS patients demonstrate only a mild reduction (~40%) in plasma levels and protease inhibitory activity is only slightly less than that of ATM [60]. Although ATS forms polymers *in vitro*, the rate constant for this process is only slightly higher than that of ATM (note, heating to ~50°C will induce wild-type serpins to polymerize *in vitro*), and markedly less than that of ATZ [44, 45]. In cell lines, secretion of ATS is delayed but not as severely as that observed with ATZ [61]. Clinically, the ATS allele only increases the risk of liver or lung disease when it is transmitted as a compound heterozygote with ATZ (Pi*SZ) [62].

For comparison, we also included 2 null alleles, Q0_hong kong_ (*S319RfsTer16*, NHK) [63], and Q0_saarbrucken_ (*E363RfsTer14*, Saar) [64]. Both null alleles yield truncated proteins. However, they are efficiently degraded intracellularly and typical of null alleles, are only associated with loss-of-function phenotypes [32, 65, 66].

As we had done with ATZ, we used a transgenic approach to generate *C*. *elegans* strains expressing the different AT mutations (Fig 1B) [43, 49]. Each quadripartite transgene contained (5’ to 3’): the *nhx-2* promoter (P_*nhx-2*_), a signal peptide fused to GFP (sGFP), an AT cDNA with the desired mutation and 4 synthetic introns and the *unc-54* 3’ UTR (Fig 1B). The P_*nhx-2*_ promoter drives intestinal cell expression, which in *C*. *elegans* subsumes the synthetic and detoxification functions of human hepatocytes [67].

**Fig 2:**
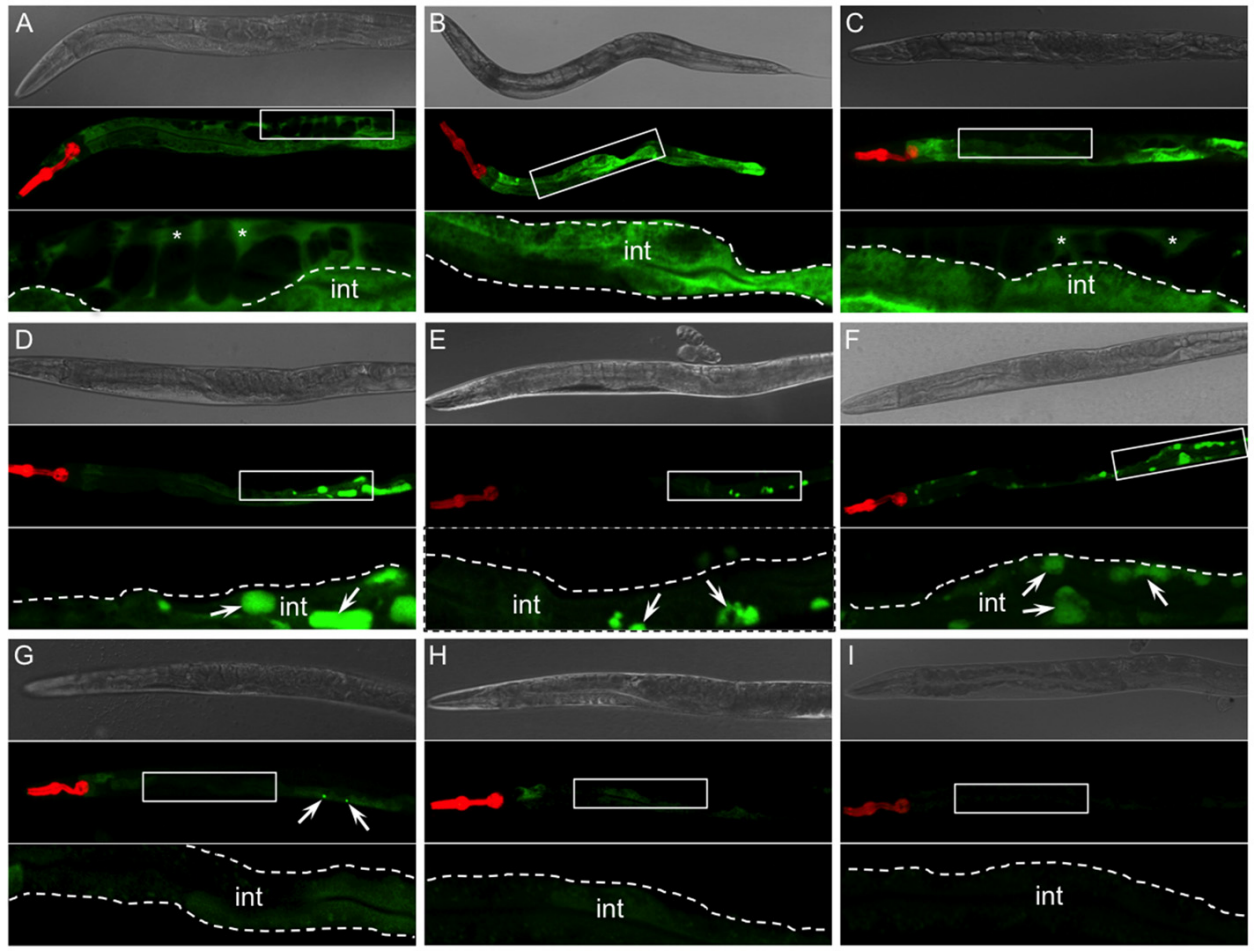
Confocal images of AT variant lines. Representative images of adult animals expressing various AT transgenes. Each panel shows a widefield DIC image of the entire worm (upper panel), corresponding fluorescence image (middle panel), and 2.5 x magnification of the region outline by a white box (lower panel, *inset*). Animals expressing the sGFP control protein efficiently secrete GFP out of the intestine (int) into the pseudocelomic space (A, *asterisks*). However, animals expressing sGFP::KDEL (ER-retention signal) control retain GFP in the ER (B).sGFP::ATM is efficiently secreted into the pseudocoelomic space (C, *asterisks*) similar to that observed in sGFP expressing controls. Animals expressing the deficient alleles, sGFP::ATZ (D), sGFP::Siiyama (E) and sGFP::Mmalton (F) accumulate protein in the ER as large globules (*arrows*) and show no evidence of secretion. Animals expressing sGFP::ATS accumulate very low steady-state levels of fusion protein with an occasional small globule detected near the tail (G, *arrows*). Animals expressing the null alleles, sGFP::Saar (H) and sGFP::NHK (I) accumulate barely detectable levels of protein in the ER.

**Fig 3:**
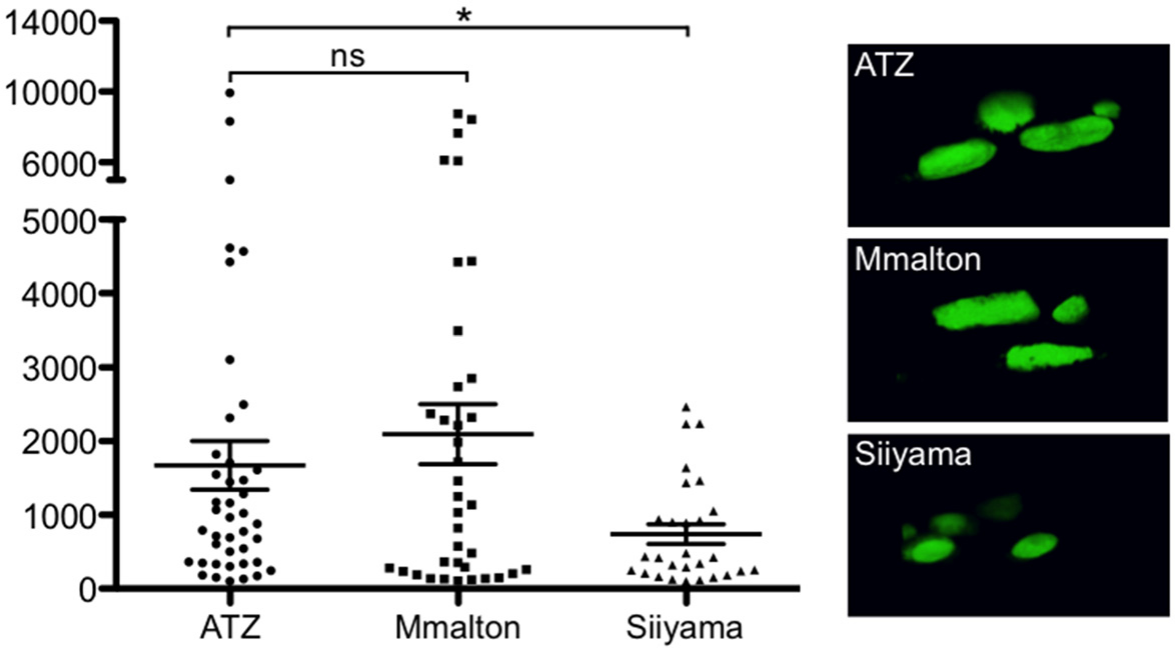
Quantification of aggregate size. Aggregate sizes accumulating in animals expressing deficient alleles. GFP-positive globules were imaged using confocal microscopy and rendered in 3D. Aggregate volumes were calculated using Volocity software. Representative images of globules from sGFP::ATZ, sGFP::Mmalton and sGFP::Siiyama are shown. Statistical significance of average size compared against ATZ was determined using two-tailed student's *t*-test, with the probabilities of results reported as *P<0.05. ns, not significant.

Transgenic lines were generated by microinjecting a P_*nhx-2*_ *sGFP*::*AT* construct with the pharyngeal co-expression marker, P_*myo-2*_ *mRFP* (the latter construct generates “red heads” which are used to select visually for the presence of transgenic animals) [49]. After transgenic strains were selected, the extrachromosomal array was integrated by irradiation and background mutations were eliminated by 6 outcrossings to the N2 strain [49]. Confocal images of representative integrants for each transgenic line were obtained (Fig 2). As shown previously [43, 49], animals expressing sGFP showed secretion into the pseudocoelomic space (Fig 2A, inset, *asterisks*). In contrast, sGFP fused to the ER retention signal, KDEL (sGFP::KDEL) [68], showed a reticular pattern within the cytoplasm, but no evidence of secretion (Fig 2B). Also as shown previously [43, 49], but re-imaged for comparison to the AT variants, sGFP::ATM and sGFP::ATZ animals showed secretion (Fig 2C) and cytoplasmic retention (Fig 2D), respectively. Specifically, sGFP::ATM expression was evident throughout the pseudocoelomic space (Fig 2C, inset, *asterisks*), whereas sGFP::ATZ showed distinct aggregates within the cell (Fig 2D, inset, *arrows*). We did not detect sGFP::ATZ secretion. The transgenic lines expressing sGFP::Siiyama (Fig 2E) and sGFP::Mmalton (Fig 2F), showed patterns similar to that of sGFP::ATZ (Fig 2D). These lines displayed characteristic GFP^+^ inclusions within intestinal cells (*arrows*), and there was no evidence of secretion. Interestingly, the sGFP::Siiyama intracellular inclusions were consistently smaller in size than those of either sGFP::ATZ or sGFP::Mmalton as assessed quantitatively by confocal microscopy (Fig 3). *C*. *elegans* expressing sGFP::ATS showed an expression pattern different from those of the polymerization mutants (Fig 2G). sGFP::ATS showed a diffuse reticular cytoplasmic pattern without evidence of secretion. An occasional cytoplasmic aggregate was also detected (Fig 2G, *arrows*). Expression of sGFP::Saar (Fig 2H) and sGFP::NHK (Fig 2I) was only detectable at high magnification. There was no evidence of intracellular aggregation or secretion of the truncated proteins.

The observations for the sGFP::NHK and sGFP::Saar transgenes were somewhat surprising since the *nhx-2* promoter has yielded consistent and robust intestinal cell expression [43, 69]. The results with the null alleles could be due to nonsense-mediated decay (NMD), low protein expression levels, efficient degradation of these truncated proteins or decreased transcription (*vide infra*). Since the frame-shifts and premature stop codons were located in the final exons of the transgenes, NMD was unlikely to play a role [70, 71].

**Fig 4:**
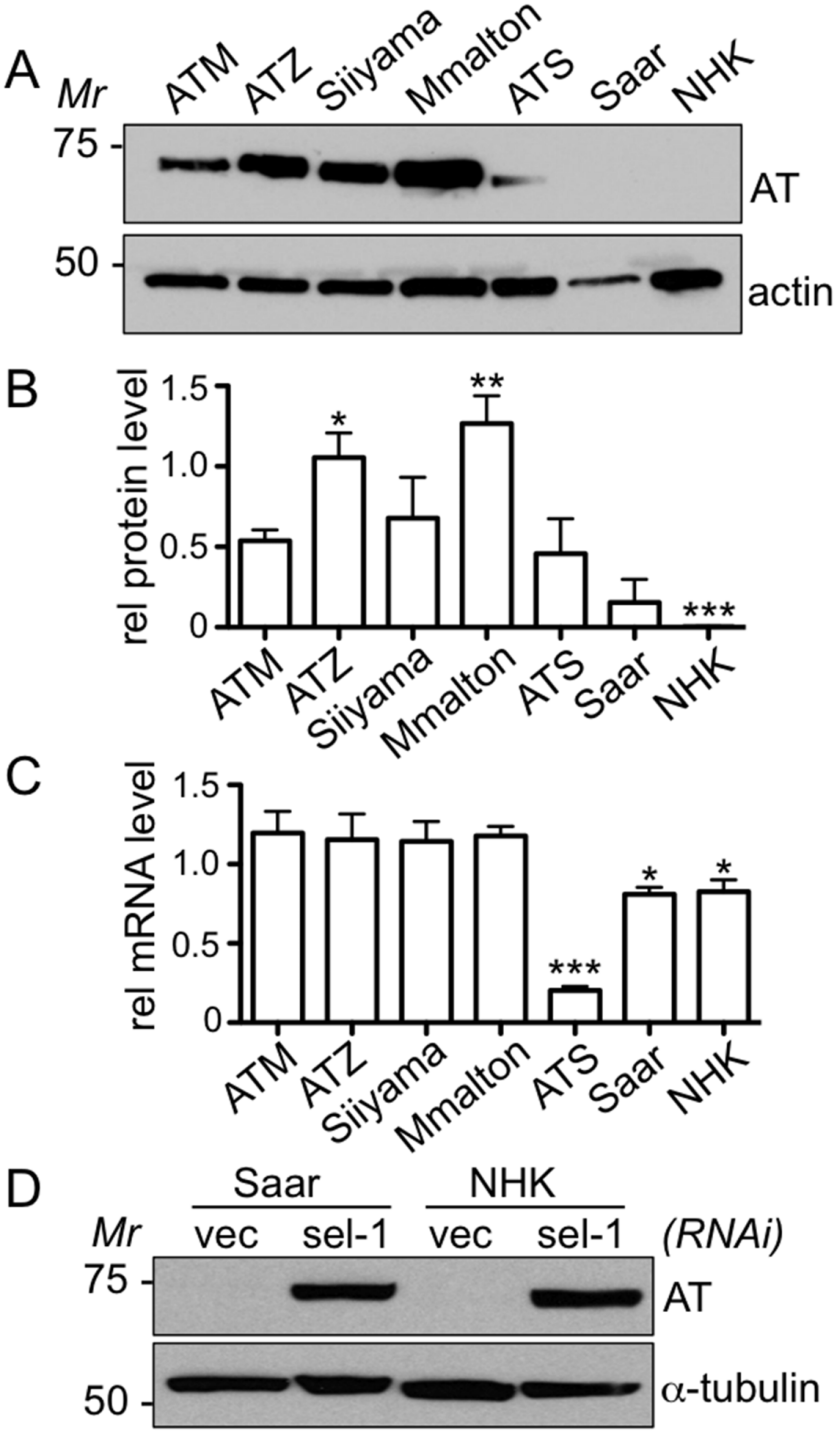
Immunoblots of AT variant transgenic lines. Western blot analysis of lysates from animals expressing different AT variant transgenes. The steady-state levels of WT and mutant sGFP::AT fusion proteins under denaturing conditions (A, *upper panel*). Actin serves as a loading control (A, *lower panel*). Relative AT protein levels as determined by densitometry (B). Statistical significance was determined using two-tailed student's *t*-test, comparing each AT variant line to ATM. Relative mRNA levels as determined by qPCR (C). Statistical significance was determined using two-tailed student's *t*-test, comparing each AT variant line to ATM. Lysates from null mutants sGFP::Saar and sGFP::NHK, exposed to *vector(RNAi)* or *sel-1(RNAi)* probed with GFP (D, *upper panel*) or α-tubulin (D, *lower panel*).

To obtain further insight into steady-state protein levels and to determine if fusion proteins of the correct molecular mass were we being synthesized for all of the AT variants, we performed semi-quantitative immunoblotting on whole animal lysates. In a representative experiment, steady-state levels of sGFP::ATZ, sGFP::Siiyama, and sGFP::Mmalton were comparable when normalized to their actin controls (Fig 4A). This trend was still evident when we average the results from 4 individual blots (Fig 4B). All of variants containing polymerization-prone mutants appeared to be slightly increased in comparison to sGFP::ATM, which was consistent with the microscopy data, and the fact that the majority of the ATM protein was efficiently secreted, whereas the mutant proteins were retained inside cells. Steady-state levels of sGFP::ATS were consistently less than those of the other variants and that of the wild-type protein, sGFP::ATM (Fig 4A and 4B). Since the S mutation results in only a partial reduction of circulating levels of the inhibitor in humans [60], and only a modest increase in the polymerization rate *in vitro* [44, 45], the low levels of sGFP::ATS observed by immunoblotting and microscopy using several different integrated transgenic lines (not shown) may reflect a low-level secretion (not detected by microscopy), enhanced intracellular degradation, or decreased transcription (*vide infra*). Although unlikely, we cannot preclude the possibility that ATS was inherently toxic to *C*. *elegans* and strains were self-selected for lower levels of baseline expression. All of the full-length AT variants were of the correct molecular mass. Consistent with the imaging data, sGFP::NHK and sGFP::Saar were not detectable by immunoblotting (Fig 4A and 4B).

To determine if steady-state mRNA levels correlated with the protein levels, we performed qRT-PCR on RNA extracts from the transgenic lines. Remarkably, all of the steady-state mRNA levels were comparable, except for that of the sGFP::ATS expressing strain (Fig 4C). This decreased mRNA level probably accounts for relatively lower levels of sGFP::ATS protein observed by fluorescence microscopy (Fig 2) and immunoblotting (Fig 4A and 4B).

### Proteostasis pathways involved AT variant protein elimination

**Fig 5:**
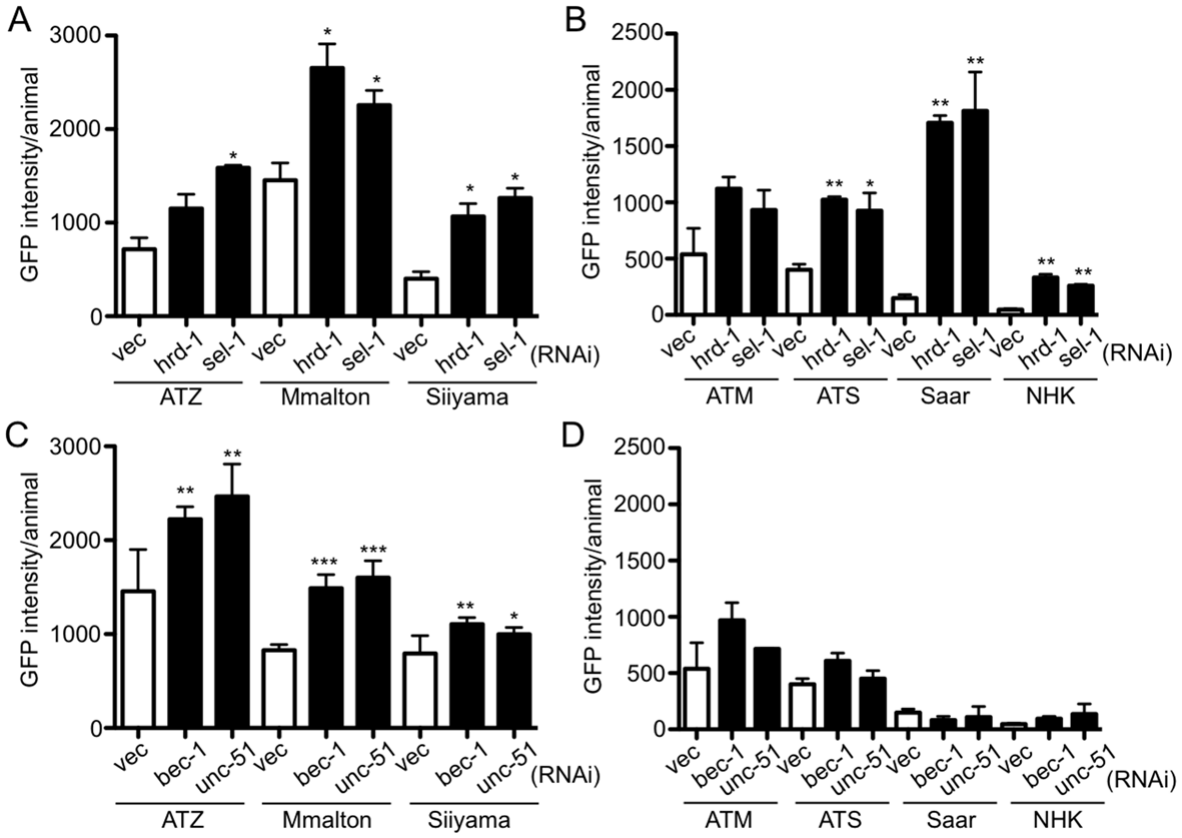
Proteostasis pathways used to clear AT variant proteins. Effect of ERAD (*hrd-1* or *sel-1*) RNAis on clearance of deficient (A) and null variants (B). Effect of autophagy (*bec-1* or *unc-51*) RNAis on clearance of deficient (C) and null variants (D). The data is representative of 6 experiments. Statistical significance was determined by comparing treatments to their respective *vec(RNAi)* controls using an unpaired, two-tailed students *t*-test, *P<0.05, **P<0.01, and ***P<0.001.

Our previous study in *C*. *elegans* [43] and those in mammalian and yeast systems suggest, in general, that the ER-associated degradation-ubiquitin proteasomal system (ERAD-UPS) and (macro)autophagy pathways are involved in the elimination of soluble misfolded monomers/oligomers [72, 73] and higher order polymers and aggregates of ATZ [72, 74, 75], respectively. To determine whether these pathways were involved in the elimination of the AT variants, we first performed RNAi on two major components of the ERAD HRD-1 E3 ligase complex, SEL-1 and HRD-1. These proteins are involved in the retro-translocation and ubiquitinylation of misfolded ER proteins as they are re-directed to the cytosol for degradation by the UPS. If this pathway was critical for clearance, then knockdown of these elements by *HRD-1(RNAi)* or *SEL-1(RNAi)* should result in accumulation of the mutant proteins. As shown previously [43], but repeated for comparison, sGFP::ATZ elimination was partially impaired by *ERAD(RNAi)* but not the *vector(RNAi)* control (Fig 5A). *ERAD(RNAi)* also partially blocked the elimination of the other aggregation prone mutants, sGFP::Mmalton and sGFP::Siiyama, as compared to controls (Figs 5A and S1). sGFP::ATS was also apparently degraded by ERAD-UPS (Fig 5B). Interestingly, both truncation mutant proteins, sGFP::Saar and sGFP::NHK, showed a marked increase over their barely perceptible baseline protein expression levels (Fig 5B). This result was consistent with mammalian cell line studies [76], and suggested that the failure to detect these proteins by fluorescence microscopy and immunoblotting was due to their efficient elimination by ERAD-UPS and not to inefficient transgene expression (Fig 4C). Indeed, immunoblots performed on protein lysates derived from animals ~48h after feeding *SEL-1(RNAi)*, showed steady state levels comparable to those of the α-tubulin controls (Fig 4D). To determine if a null AT mutant was efficiently eliminated by baseline or enhanced ERAD-UPS activity in *C*. *elegans*, we co-injected the unfolded protein response (UPR) sensor, the BiP/HSP-4 promoter fused to mCherry (P_hsp-4_ *mCherry*) [77], into the sGFP::NHK expressing animals. HSP-4 was constitutively activated (S2 Fig), indicating that UPR activation of ERAD-UPS was most likely responsible for the efficient elimination of sGFP::NHK. UPS activation was observed also in all of the strains carrying deficient alleles. However, since the P_hsp-4_ *mCherry* transgene was propagated as an extrachromosomal array, quantitative comparisons assessing the magnitude of UPR activation were not feasible.

We next examined the role of autophagy on AT variant clearance. As shown previously [43], but repeated as a control, RNAi of two essential autophagy genes, UNC-51 (orthologue of the *S*. *cerevisiae* autophagy protein Atg1p) and BEC-1 (orthologue of yeast and mammalian proteins Atg6/Vps30/Beclin1) enhanced the accumulation of sGFP::ATZ (Fig 5C). Similarly, sGFP::Mmalton and sGFP::Siiyama were eliminated partially by autophagy (Fig 5C). In contrast, autophagy appeared to have no significant effect on the steady state protein levels of sGFP::ATS, sGFP::Saar or sGFP::NHK (Fig 5D).

### Effects of AT variants on health and longevity

**Fig 6:**
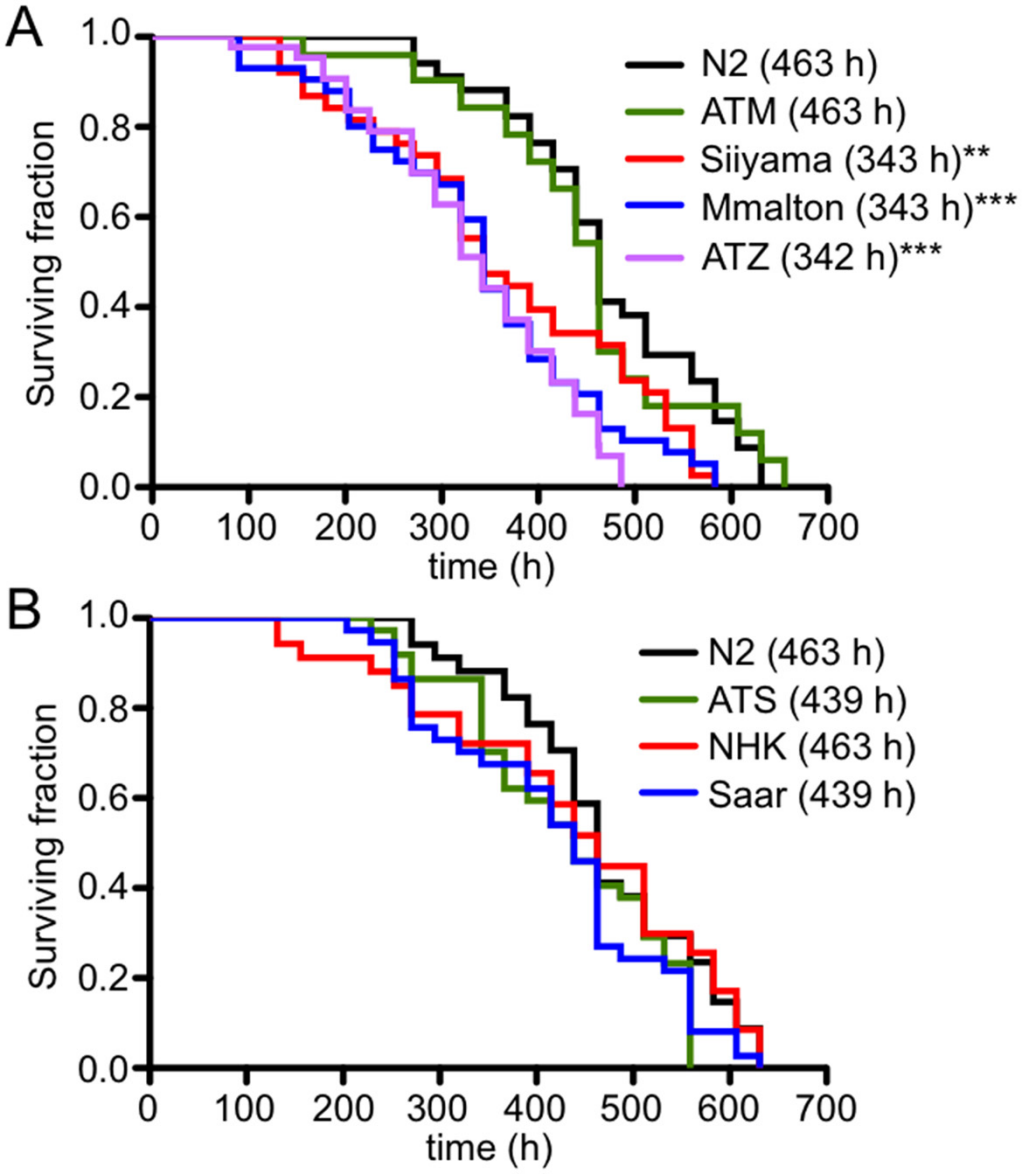
Effect of AT variant protein expression on longevity. Representative Kaplan-Meier curves of deficient mutants (A). N2 (*black*), ATM (*green*), Siiyama (*red*), Mmalton (*blue*) and ATZ (*lavender*). Median survival times (in *parenthesis*). Representative Kaplan-Meier curves of ATS and null alleles (B). N2 (*black*), ATS (*green*), NHK (*red*) and Saar (*blue*). Statistical significance determined using the Log-Rank (Mantel-Cox) test, with probabilities of results reported as **P<0.001, or ***P<0.0001.

In our previous study, the accumulation of sGFP::ATZ had a deleterious effect on the animals as manifest by a decrease in longevity, slow larval growth and smaller brood sizes [43]. To determine whether expression of the other AT variants were also proteotoxic to *C*. *elegans*, we measured their lifespan by plotting Kaplan-Meier curves [78]. Animals expressing sGFP::ATM showed overall longevity and a median survival time comparable to those of wild-type (N2) animals (Fig 6A). This result confirmed that wild-type AT, which is a *bona fide* peptidase inhibitor, was not toxic to the animals. In contrast, animals expressing the polymerization mutants, sGFP::Siiyama and sGFP::Mmalton, showed shorter lifespans and median survival times as compared to those expressing sGFP::ATM (Fig 6A). These results were similar to those previously published for the aggregation-prone mutant, sGFP::ATZ, which were repeated here to permit direct comparison with the other strains [43]. Animals expressing sGFP::ATS, sGFP::Saar and sGFP::NHK showed relatively normal lifespans and median survival times when compared to the wild-type N2 strain (Fig 6B).

**Fig 7:**
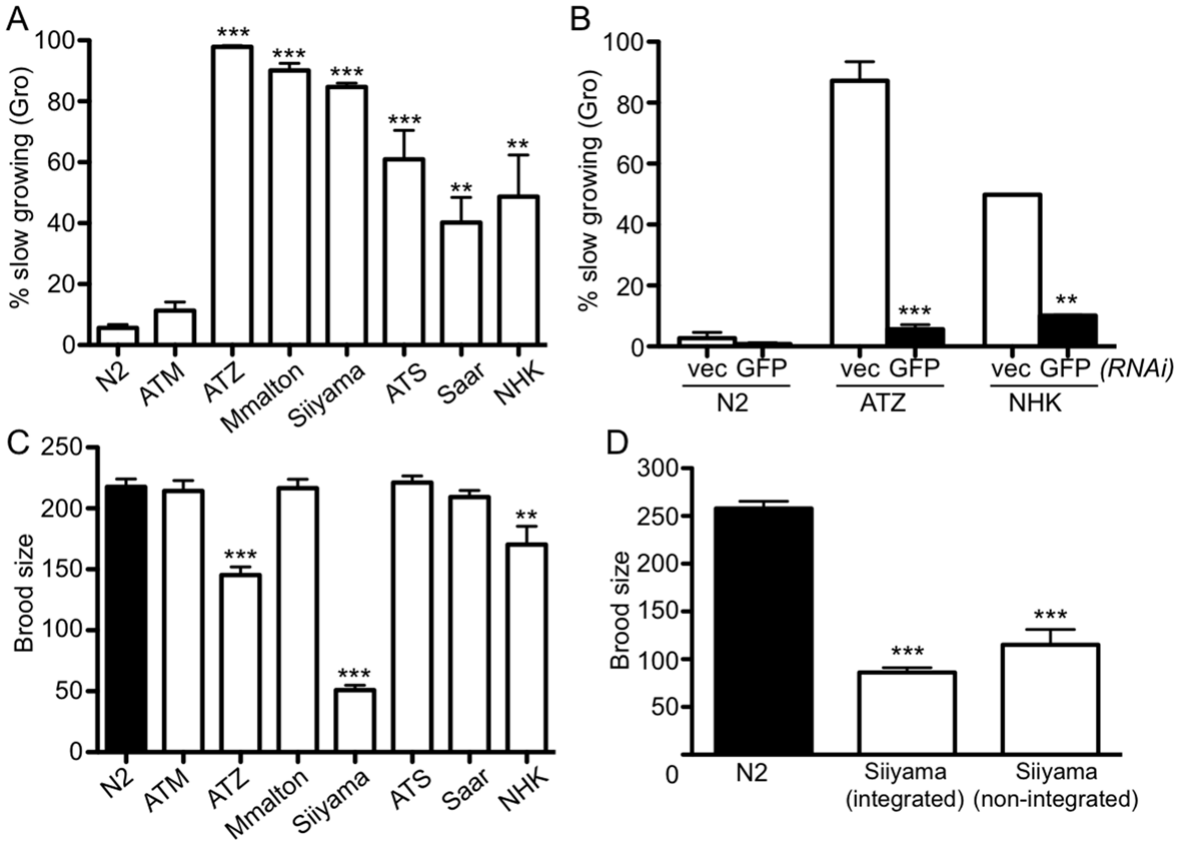
Effect of AT variant protein expression on larval growth and brood sizes. Assessment of slow growth (Gro) phenotype during post-embryonic development (A). Statistical significance was determined comparing Gro phenotypes of AT variants to wild-type ATM-expressing lines using an unpaired, two-tailed students *t*-test. Reversal of Gro phenotype by *GFP(RNAi)* (B). Statistical significance was determined by comparing treatments to their respective *vec(RNAi)* controls using an unpaired, two-tailed students *t*-test. Brood size assessment of AT variant lines (C). Statistical significance was determined comparing brood size of AT variants compared to wild-type ATM-expressing lines using an unpaired, two-tailed students *t*-test. Brood size comparison between integrated and non-integrated Siiyama expressing lines (D). Statistical significance was determined comparing brood size of AT variants was compared to wild-type ATM-expressing lines using an unpaired, two-tailed students *t*-test. In all experiments, the probabilities of results were reported as *P<0.05, **P<0.01, or ***P<0.001.

We next determined whether any of the AT variants affected larval growth during post-embryonic development (Gro phenotype). While expression of sGFP::ATM had no significant effect on post-embryonic growth, all of the AT variants demonstrated a marked delay (Fig 7A). Over 80% of the animals expressing one of the three polymerizing mutants, sGFP::ATZ, sGFP::Mmalton or sGFP::Siiyama were growth restricted (Fig 7A). Approximately 60% of animals expressing sGFP::ATS also showed the Gro phenotype (Fig 7A). Surprisingly, ~40–50% of animals expressing the null AT mutants, sGFP::Saar or SGFP::NHK, exhibited the Gro phenotype (Fig 7A). This latter result was surprising since null AT mutants are not typically associated with a gain-of-function phenotype. The Gro phenotype in the null mutants was not due to serpin over-expression *per se*, as the sGFP::ATM-expressing line had a minimal effect on development. To verify that this Gro phenotype was variant-induced, and not secondary to a transgene integration event or a background mutation, AT protein expression was knocked-down in ATZ and NHK animals by hatching the animals on bacteria expressing GFP dsRNA. *GFP(RNAi)* treatment completely reversed the Gro phenotype (Fig 7B). We concluded that the Gro phenotype was correlated with expression of the mutant proteins and not a secondary genetic mutation.

Another general measure of animal well-being is the ability to reproduce and generate normal brood sizes. As shown previously [43], and reproduced to provide a basis for comparison, sGFP::ATZ-expressing animals had significantly reduced brood sizes relative to sGFP::ATM-expressing animals or wild-type (N2) controls (Fig 7C). Only sGFP::Siiyama-, and to a much lesser extent, sGFP::NHK-expressing animals showed reduced brood sizes (Fig 7C). The decreased brood sizes in sGFP::Siiyama-expressing animals was striking, and much lower than that observed with sGFP::ATZ expressing animals. This result was consistent with our general observation that the sGFP::Siiyama-expressing animals were smaller and more sickly appearing than the sGFP::ATZ expressing animals. This “sickly” phenotype was observed with different sGFP::Siiyama-expressing transgenic lines (S3 Fig). However, to verify the extremely low brood sizes in the sGFP::Siiyama animals was not a coincidental effect associated with different transgene integration sites or a stochastic irradiation-induced mutation not eliminated by outcrossing, we repeated the brood size analysis using animals propagating a non-integrated, extrachromosomal transgene array. These latter animals also showed a significant decrease in brood (Fig 7D) suggesting that the misfolded Siiyama species was particularly proteotoxic to *C*. *elegans*.

## Discussion

There were two major objectives of this study. The first was to determine the capacity of different AT variants associated with ATD to induce a cellular gain-of-function proteotoxic phenotype. The second was to assess the ability of a previously established *C*. *elegans* ATD model to screen for evidence of AT-induced cellular toxicity [43]. The use of a *C*. *elegans* model as a screening tool has the advantage over other transgenic systems in that they offer multiple quantifiable physiological read-outs and strain generation is relatively rapid and inexpensive [42]. AT variants are typically characterized as either deficient or null alleles based on low or negligible circulating levels of the serpin, respectively [29, 32, 34–36]. In this study, we analyzed three representative deficient (Mmalton, Siiyama, and ATS) and two null (NHK and Saar) alleles and compared these results with the most prevalent deficient allele, ATZ [7, 13, 79–81]. From a clinical perspective; ATZ and Mmalton are associated with protein misfolding, ER retention and aggregation (hepatic inclusions), intracellular degradation by autophagy and ERAD, and a gain-of-function proteotoxicity causing liver disease [52–58]. Although Siiyama can lead to hepatic inclusions [82], too few patients have been analyzed to determine whether homozygotes with this allele also sustain liver damage. In general, the three aggregation-prone mutants (ATZ, Siiyama and Mmalton) had the same general effects in *C*. *elegans*: the intracellular accumulation of misfolded proteins and proteotoxicity manifested by abnormalities in three general indicators of animal well-being (egg laying and hatching of viable larvae, time required for larvae to develop into adults and longevity). Although protein expression levels were comparable by immunoblotting, and we examined several different integrated transgenic lines, it is difficult to determine whether Siiyama or Mmalton is more toxic than ATZ. Moreover, since the phenotypic studies required propagating strains, we may have selected for animals that expressed amounts of the serpin that were inherently less toxic. Nonetheless, among the three most toxic aggregation-prone deficient alleles, the Siiyama mutants had the smallest brood sizes and were sicklier (smaller and thinner). In addition, the intracellular aggregates were smaller in size. The mechanism of serpin polymerization has been a subject of some debate. However, the crystal structure shows a domain-swapping mechanism, likely induced by a delay in folding by the E342K mutation. Rapid folding of the wild-type protein bypasses the global free-energy minimum for the metastable serpin and facilitates reactive site loop exposure rather than A-sheet insertion [6]. As structures for polymerized Siiyama or Mmalton do not exist, it is not clear whether the elegant mechanism for ATZ polymerization applies to shutter domain mutants as well. Thus, these observations raise the intriguing possibility that different mutations lead to aggregates with different structural properties, and based on the nature of exposed domains, higher order structures and interacting species capable of inducing different types of proteotoxicity. This hypothesis is supported by studies of the synucleinopathies, where specific pathological features correlate with different forms of α-synuclein (including the A30P mutant) that associate into distinct structural assemblies [83]. Alternatively, the Siiyama mutants may be less prone to developing large aggregates, thereby leading to the generation of more monomers and oligomers, which are considered to be the toxic species in many conformational disorders [84]. It should be noted, however, data on toxic oligomeric species are related to proteins that localize to the cytosol or the extracellular space. We are unaware of any studies extending these observations to proteins in the ER lumen. Interestingly, mutations in neuroserpin/SERPINI1 induce a conformational disease in neurons leading to a form of dementia [85, 86]. Molecular modeling of different types of SERPINI1 mutations on the serpin scaffold predict a hierarchy of mutations, where those that predict a greater degree of conformational instability correlate with more severe disease (e.g., earlier onset), greater involvement in the number of neurons and an increase in the number and size of intracellular inclusions [87]. While disease modifiers will affect disease penetrance and expressivity, the combined data on serpinopathies suggest that quantitative and qualitative features of the serpin polymer may differentially affect the host cells proteostasis response and degree of toxicity. *C*. *elegans* has the potential of teasing apart these differences by systematically modifying different components of the proteostasis pathways and assessing the animals’ response to different AT variants.

Negligible circulating levels of AT and the resultant risk for emphysema is the major loss-of-function phenotype associated with homozygous null AT mutants [32, 65, 66]. Since many of the null mutants are due to point mutations and indels leading to frame shifts and premature stop codons, the resultant truncated proteins (if they are synthesized) are retained transiently in the ER and then efficiently degraded by ERAD [63, 64, 88]. We are unaware of any documented patients with homozygous null mutations leading to a gain-of-function phenotype such as liver disease. In contrast to the deficient alleles, many of the truncated proteins that lack key structural elements within the serpin scaffold are likely to fold improperly and trigger an exuberant UPR [34, 63, 72, 89]. Indeed, Saar and NHK proteins were both efficiently degraded by ERAD in *C*. *elegans*. However, we were surprised to find these proteins, especially NHK, yielded a gain-of-function phenotype manifested by decreased fecundity and growth; albeit less than that of the aggregation-prone deficient alleles. While this gain-of-function phenotype could be attributed to over-expression of any heterologous protein in a transgenic system, it was not observed with the ATM controls, which generated at steady-state, greater amounts of protein. Moreover, since Saar and NHK do not form visible intracellular inclusions typical of aggregation-prone proteins and do not depend on the autophagy pathway for elimination, the mechanism of toxicity is likely different from than that induced by ATZ, Mmalton and Siiyama. We suggest that the homozygosity of the inbred N2 strain along with its relatively short lifespan (2–3 weeks) and controlled laboratory environment, may have revealed a phenotype not readily apparent in the small numbers of human patients with null alleles. Moreover, the human genetic heterogeneity combined with multiple environmental confounders may make assessments for null-specific phenotypes more difficult to discern [63]. Nonetheless, these results prompt us to consider whether patients homozygous for some null AT alleles may be at risk for hepatocellular injury under certain environmental factors (e.g., viral hepatitis, ethanol consumption). In support of this notion, chronic ER stress due to constitutive synthesis of misfolded proteins such as CFTR (ΔF508) or ATZ lead to a maladaptive stress response that is deleterious to the cell [90]. Since expression of all the AT variants led to some degree of chronic UPR activation, including the null variants, overall proteostasis may falter leading to decreased reproductive capacity, slower growth and decreased lifespan. Loss of effective proteostasis appears to be a major contributor to the aging process as well [91].

The ATS deficient allele appears to be of little clinical significance unless it is inherited as a compound heterozygote along with ATZ [62]. Protein levels, even in Pi*SS patients do not fall below sub-therapeutic levels, and the serpin serves as a normal peptidase inhibitor. While ATS polymerizes *in vitro*, the rate constant for this reaction is marginally greater than that of the wild-type protein control. Consistent with the clinical observation, animals expressing ATS only showed a mild slow growth phenotype, but a normal lifespan. In terms of its disposal, ATS was eliminated by ERAD, with autophagy playing no detectable role. Thus, like in humans, ATS-expressing *C*. *elegans* followed a milder course, rather than those expressing the more toxic deficient alleles. However, these results should be tempered by the inability to select for a strain whose steady-state protein and mRNA levels for ATS were comparable to the other deficient allele-expressing strains. We cannot readily explain this discrepancy as DNA sequence of the ATS transgene differs from that of ATM by a single nucleotide and the DNA sequences of the all the transgenes were repeatedly verified. Since relatively low-level expression of sGFP::ATS still showed a slow-growth phenotype, it is conceivable that higher levels of expression were more toxic and eliminated the selection process. In general, however, these data suggested that the *C*. *elegans* model was efficient in discriminating between different types of AT variants by successfully modeling the severity of their clinical phenotypes.

Modeling of human diseases in *C*. *elegans* has distinct advantages over other types of model systems including robust genetic technologies and a transparent body that permits the tracking of tissues and fluorescently tagged molecules in real time [42]. However, an animal model is limited if the phenotypes do not accurately portray the fundamental pathogenicity observed in human tissues. Fortunately, *C*. *elegans* has proven to be extremely useful in the modeling of human conformational diseases where the misfolded species accumulates in the ER or the cytosol [43, 92–99]. This success is related in part to the conservation of the basic proteostasis machinery between nematodes and higher vertebrates [100, 101]. For this reason, we modeled the gain-of-function phenotype of ATD in *C*. *elegans* by generating transgenic animals synthesizing and accumulating ATZ [43, 49, 50]. These mutants have proven useful in two major ways. First, they can be incorporated into high-throughput, high content drug discovery platforms designed to identify compounds that block or eliminate the accumulation of the toxic species [49]. Second, they can be used in genome-wide screens for genetic modifiers and potential drug targets of the disease phenotype [50]. The data from this report described a third utility: the rapid and economical means for screening of disease variants. As whole genome and exome sequencing becomes more affordable, genomic DNA from patients with undiagnosed and rare diseases is being analyzed to decipher the genetic causes of these disorders. In many cases, it may be difficult to determine whether a sequence variation represents a true gain- and/or loss-of-function mutation or simply a benign polymorphism. One means to differentiate among these possibilities is to express the gene in cell lines or model organisms and assess for phenotypic change. We suggest that for some types of proteins, *C*. *elegans* may be the system of choice due to their relatively inexpensive experimental costs, ease in genetic manipulation, and the ability to examine multiple phenotypes in a complex multicellular organism with a short lifespan. We find this strategy especially true if a model is already established. In the case ATD, assessing the toxicity of many of the >120 AT mutations is now imminently feasible.

## Supporting Information

S1 Fig Effect of ERAD and Autophagy RNAis on AT clearance. Effect of ERAD (*hrd-1* or *sel-1*) RNAis on clearance of deficient (A) and null variants (B). Effect of autophagy (*bec-1* or *unc-51*) RNAis on clearance of deficient (C) and null variants (D). The data is same as that shown in Fig 5 except that the *y*-axis is represented as fold-change in GFP relative to *vec(RNAi)* control. Statistical significance was determined by comparing treatments to their respective *vec(RNAi)* controls using an unpaired, two-tailed students *t*-test, *P<0.05, **P<0.01, and ***P<0.001.
(TIF)

S2 Fig Induction of UPR by NHK expression. sGFP::NHK animals were crossed with animals expressing the UPR reporter, P_*hsp-4*_::mCherry. Hsp-4::mCherry expression was then assessed in homozygous F2 progeny. Statistical significance was determined by comparing Hsp-4::mCherry expression in N2 and NHK animals using an unpaired, two-tailed students *t*-test. Brood size assessment of AT mutant lines (C). Brood size comparison between integrated and non-integrated Siiyama expressing lines (D). The probabilities of results were reported as *P<0.05, **P<0.01, and ***P<0.001.
(TIF)

S3 Fig sGFP::Siiyama expressing lines appear more sicklier. Representative DIC images of 2-day old adults expressing sGFP::Siiyama (A). Animals are smaller in size and lack the dark intestinal region seen in normal, healthy animals (A, *upper panel*). The sickly appearance can be completely reversed by knocking down sGFP::Siiyama expression via *GFP(RNAi)* (A, *lower panel*). sGFP::Siiyama-expressing animals accumulate fewer eggs in utero (B). Representative DIC images of the uterus of animals expressing sGFP::ATM (B, *upper panel*), sGFP::ATZ (B, *middle panel*) and sGFP::Siiyama (B, *lower panel*). Note, the presence of fewer eggs *in utero* of sGFP::Siiyama animals (B, *lower panel*, *arrows*). Quantification of the number of eggs *in utero* (C). Statistical significance was determined using an unpaired, two-tailed students *t*-test. The probabilities of results were reported as ***P<0.001.
(TIF)

S1 Table List of transgenic lines. Official strain names and genotypes of all transgenes used in this study are listed. del, deletion; fs, frame-shift; Ter, termination.
(DOCX)

S2 Table Mutagenesis primers. Primer names and oligonucleotide sequences are listed. All AT deficiency and null alleles were generated by site-directed mutagenesis using the wild-type ATM as the template.
(DOCX)
