# Supplementary figures and images for: Deficient and Null Variants of SERPINA1 Are Proteotoxic in a Caenorhabditis elegans Model of α1-Antitrypsin Deficiency
Source: PLoS One. 2015 Oct 29;10(10):e0141542. doi: 10.1371/journal.pone.0141542 (PMC4626213; doi:10.1371/journal.pone.0141542)

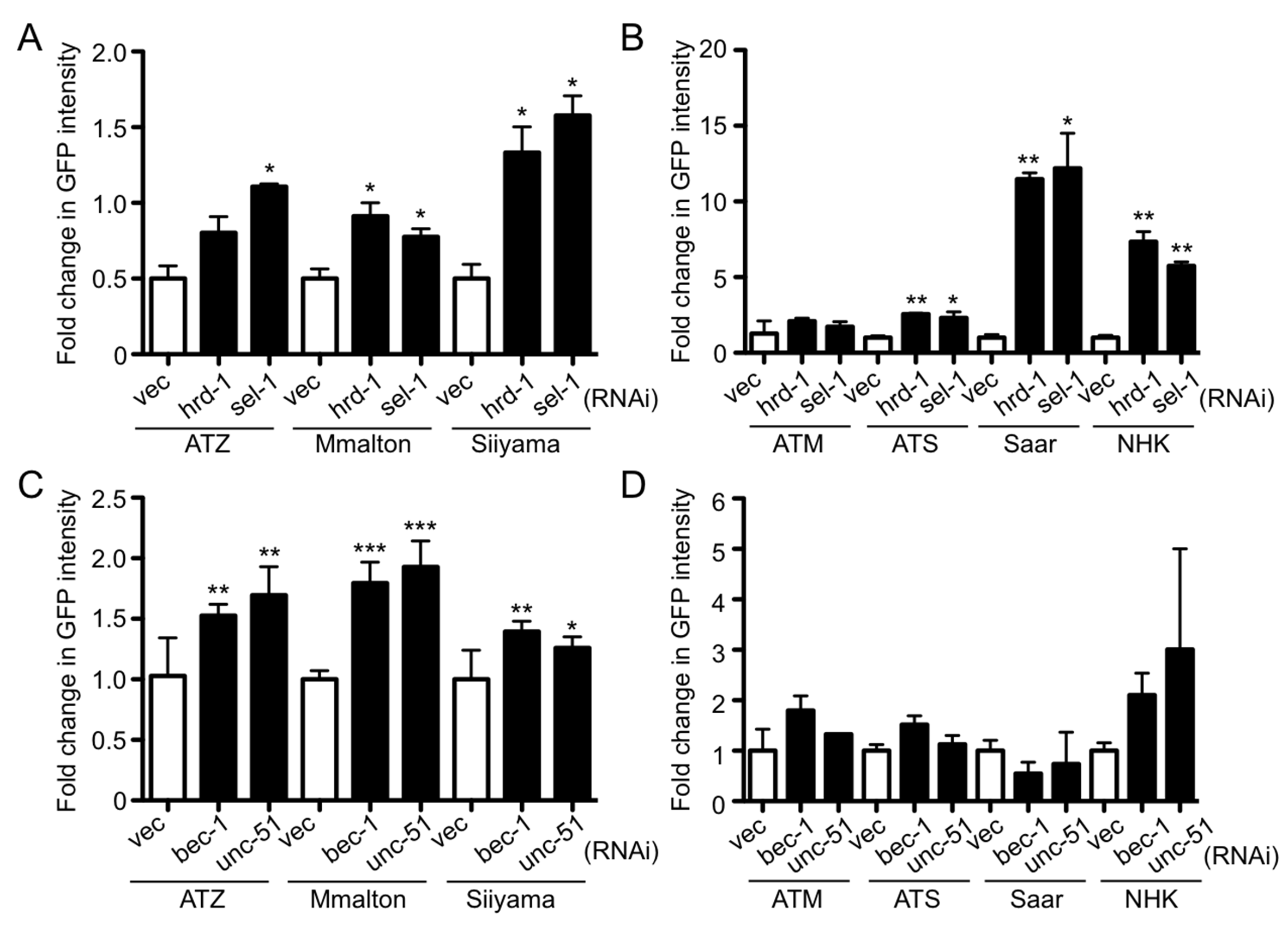

Supplement: S1 Fig — Effect of ERAD (hrd-1 or sel-1) RNAis on clearance of deficient (A) and null variants (B). Effect of autophagy (bec-1 or unc-51) RNAis on clearance of deficient (C) and null variants (D). The data is same as that shown in Fig 5 except that the y-axis is represented as fold-change in GFP relative to vec(RNAi) control. Statistical significance was determined by comparing treatments to their respective vec(RNAi) controls using an unpaired, two-tailed students t-test, *P<0.05, **P<0.01, and ***P<0.001. (TIF) [file pone.0141542.s001.tif]

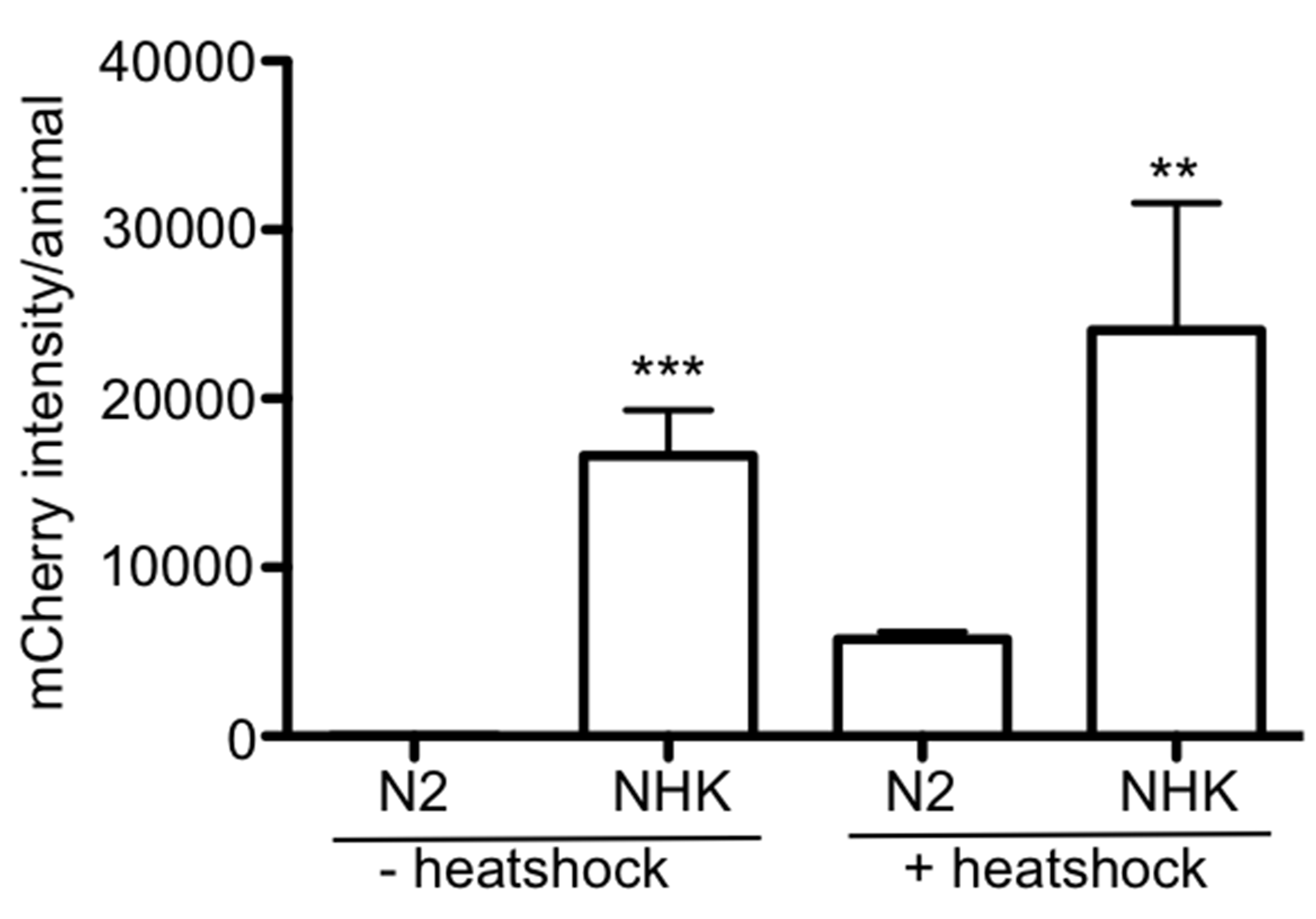

Supplement: S2 Fig — sGFP::NHK animals were crossed with animals expressing the UPR reporter, Phsp-4::mCherry. Hsp-4::mCherry expression was then assessed in homozygous F2 progeny. Statistical significance was determined by comparing Hsp-4::mCherry expression in N2 and NHK animals using an unpaired, two-tailed students t-test. Brood size assessment of AT mutant lines (C). Brood size comparison between integrated and non-integrated Siiyama expressing lines (D). The probabilities of results were reported as *P<0.05, **P<0.01, and ***P<0.001. (TIF) [file pone.0141542.s002.tif]

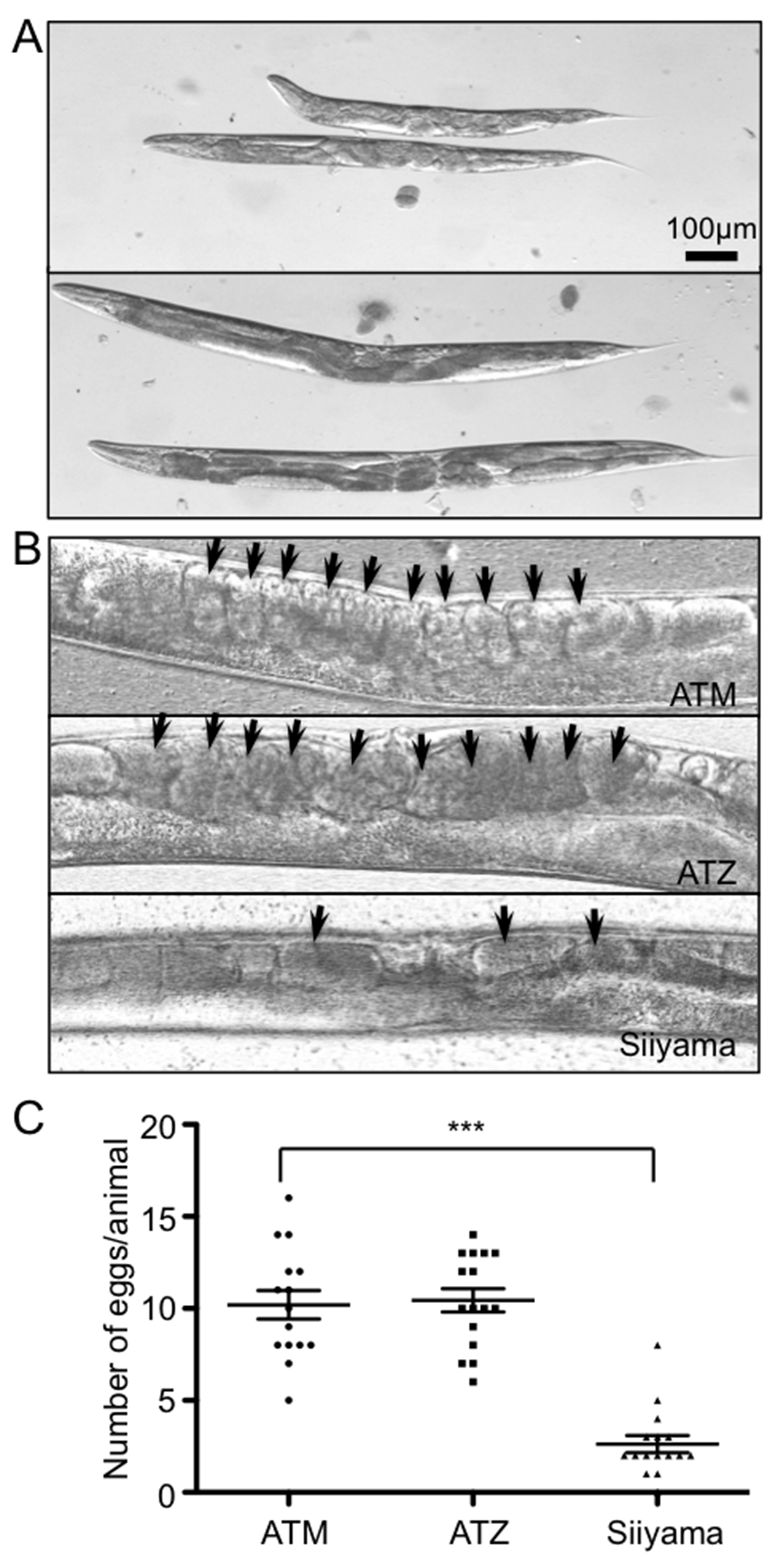

Supplement: S3 Fig — Representative DIC images of 2-day old adults expressing sGFP::Siiyama (A). Animals are smaller in size and lack the dark intestinal region seen in normal, healthy animals (A, upper panel). The sickly appearance can be completely reversed by knocking down sGFP::Siiyama expression via GFP(RNAi) (A, lower panel). sGFP::Siiyama-expressing animals accumulate fewer eggs in utero (B). Representative DIC images of the uterus of animals expressing sGFP::ATM (B, upper panel), sGFP::ATZ (B, middle panel) and sGFP::Siiyama (B, lower panel). Note, the presence of fewer eggs in utero of sGFP::Siiyama animals (B, lower panel, arrows). Quantification of the number of eggs in utero (C). Statistical significance was determined using an unpaired, two-tailed students t-test. The probabilities of results were reported as ***P<0.001. (TIF) [file pone.0141542.s003.tif]
