# Supplementary material for: Deficient and Null Variants of SERPINA1 Are Proteotoxic in a Caenorhabditis elegans Model of α1-Antitrypsin Deficiency
Source: PLoS One. 2015 Oct 29;10(10):e0141542. doi: 10.1371/journal.pone.0141542 (PMC4626213; doi:10.1371/journal.pone.0141542)
Supplement: S1 Table — Official strain names and genotypes of all transgenes used in this study are listed. del, deletion; fs, frame-shift; Ter, termination. (DOCX) [file pone.0141542.s004.docx]

S1 Table. List of transgenic lines

| Strain name | Genotype | Protein expressed | Mutation | Allele class |
| --- | --- | --- | --- | --- |
| VK2155 | vkIs2155[nhx-2p::sGFP;myo-2p::mCherry] | sGFP |  |  |
| VK2315 | vkIs2315[nhx-2p::sGFP-KDEL;myo-2p::mCherry] | sGFP-KDEL |  |  |
| VK1950 | vkIs1538[nhx-2p::sGFP::ATM;myo-2p::mCherry] | sGFP::ATM | wild-type | wild-type |
| VK1882 | vkIs1444[nhx-2p::sGFP::ATZ;myo-2p::mCherry] | sGFP::ATZ | E342K | deficiency |
| VK2050 | vkIs1902[nhx-2p::sGFP::Mmalton;myo-2p::mCherry] | sGFP::Mmalton | F52del | deficiency |
| VK2015 | vkIs1845[nhx-2p::sGFP::Siiyama;myo-2p::mCherry] | sGFP::Siiyama | S53F | deficiency |
| VK2013 | vkIs1478[nhx-2p::sGFP::ATS;myo-2p::mCherry] | sGFP::ATS | E264V | deficiency |
| VK1973 | vkIs1555[nhx-2p::sGFP::NHK;myo-2p::mCherry] | sGFP::NHK | S319RfsTer16 | null |
| VK1958 | vkIs1720[nhx-2p::sGFP::Saar;myo-2p::mCherry] | sGFP::Saar | E363RfsTer14 | null |
