# Supplementary material for: Deficient and Null Variants of SERPINA1 Are Proteotoxic in a Caenorhabditis elegans Model of α1-Antitrypsin Deficiency
Source: PLoS One. 2015 Oct 29;10(10):e0141542. doi: 10.1371/journal.pone.0141542 (PMC4626213; doi:10.1371/journal.pone.0141542)
Supplement: S2 Table — Primer names and oligonucleotide sequences are listed. All AT deficiency and null alleles were generated by site-directed mutagenesis using the wild-type ATM as the template. (DOCX) [file pone.0141542.s005.docx]

S2 Table. Mutagenesis primers

| Primer name | Primer sequence |
| --- | --- |
| ATZ.mut.F | GGCTGTGCTGACCATCGATAAGAAAGGGACTGAAGCTGC |
| ATZ.mut.R | GCAGCTTCAGTCCCTTTCTTATCGATGGTCAGCACAGCC |
| Mmalton.mut.F | CCAACAGCACCAATATCTTCTCCCGTAAGTTTAAACATG |
| Mmalton.mut.R | CATGTTTAAACTTACGGGAGAAGATATTGGTGCTGTTGG |
| Siiyama.mut.F | CCAACAGCACCAATATCTTCTTCTTCCGTAAGTTTAAACATG |
| Siiyama.mut.R | CATGTTTAAACTTACGGAAGAAGAAGATATTGGTGCTGTTGG |
| ATS.mut.F | GGAAACTACAGCACCTGGTAAATGAACTCACCCACG |
| ATS.mut.R | CGTGGGTGAGTTCATTTACCAGGTGCTGTAGTTTCC |
| NHK.mut.F | GCAATGGGGCTGACCTCCGGGGTCACAGAGG |
| NHK.mut.R | CCTCTGTGACCCCGGAGGTCAGCCCCATTGC |
| Saar.mut.F | CCCATGTCTATCCCACCTCGAGGTCAAGTTCAACAAACCC |
| Saar.mut.R | GGGTTTGTTGAACTTGACCTCGAGGTGGGATAGACATGGG |
